# Genomic Gigantism is not Associated with Reduced Selection Efficiency in Neotropical Salamanders

**DOI:** 10.1007/s00239-024-10177-w

**Published:** 2024-06-06

**Authors:** Hairo Rios-Carlos, María Guadalupe Segovia-Ramírez, Matthew K. Fujita, Sean M. Rovito

**Affiliations:** 1 Unidad de Genómica Avanzada, Centro de Investigación y de Estudios Avanzados del Instituto Politécnico Nacional, km 9.6 Libramiento Norte Carretera Irapuato-León, Irapuato, Guanajuato México; 2 Department of Biology, Amphibian and Reptile Diversity Research Center, The University of Texas, Arlington, TX USA

**Keywords:** Genome size, Relaxation of natural selection, Neotropical salamanders, Transcriptomes, Selection efficiency

## Abstract

Genome size variation in eukaryotes has myriad effects on organismal biology from the genomic to whole-organism level. Large genome size may be associated with lower selection efficiency because lower effective population sizes allow fixation of deleterious mutations via genetic drift, increasing genome size and decreasing selection efficiency. Because of a hypothesized negative relationship between genome size and recombination rate per base pair, increased genome size could also increase the effect of linked selection in the genome, decreasing the efficiency with which natural selection can fix or remove mutations. We used a transcriptomic dataset of 15 and a subset of six Neotropical salamander species ranging in genome size from 12 to 87 pg to study the relationship between genome size and efficiency of selection. We estimated d*N*/d*S* of salamanders with small and large genomes and tested for relaxation of selection in the larger genomes. Contrary to our expectations, we did not find a significant relationship between genome size and selection efficiency or strong evidence for higher d*N*/d*S* values in species with larger genomes for either species set. We also found little evidence for relaxation of selection in species with larger genomes. A positive correlation between genome size and range size (a proxy of population size) in this group disagrees with predictions of stronger drift in species with larger genomes. Our results highlight the complex interactions between the many forces shaping genomic variation in organisms with genomic gigantism.

**Supplementary Information:**

The online version contains supplementary material available at 10.1007/s00239-024-10177-w.

## Introduction

Eukaryotes vary by over three orders of magnitude in genome size or C-value (measured as the mass (pg) of DNA per haploid nucleus, with 1 pg = 978 Mpb) (Gregory and Hebert 1999; www.genomesize.com). Although gene number does show a positive relationship with genome size in eukaryotes, the magnitude of this relationship is insufficient to explain genome size increases (Lynch and Conery 2003). The largest eukaryotic genomes are found in land plants, lungfish, and salamanders and have expanded primarily as the result of repeat element proliferation (Sun et al. 2012; Pellicer et al. 2018; Meyer et al. 2021) and the two available salamander and lungfish genomes contain similar number of genes to other vertebrates with much smaller genomes (Nowoshilow et al. 2018; Meyer et al. 2021). This wide variation in genome size has myriad consequences from the genomic to whole-organism levels, with relationships between genome size and cell cycle time, tissue complexity, relative size of sensory organs, and body size in multiple groups (Hanken and Wake 1993; Roth et al. 1994; Herrick 2011; Decena-Segarra et al. 2020; Meyer et al. 2021).

Hypotheses that seek to explain genome size variation can be divided into those that propose an adaptive explanation for genome size increase, via either selection on genome size itself or on phenotypes correlated with genome size, and nonadaptive explanations. Adaptive hypotheses propose that natural selection on phenotypes associated with genome size, often through cell size-genome size correlations, lead to changes in genome size (Cavalier-Smith 1982; Vinogradov 1995). Nonadaptive hypotheses center on the fact that the integration of repeat elements, as well as other mutations, into the genome depends on the interplay of natural selection and genetic drift (Ohta 1973; Lynch et al. 2011; Jeffares et al. 2015). These hypotheses typically invoke decreased meiotic recombination rate per base pair in larger genomes, stronger genetic drift in populations with smaller effective population size (*N*_*e*_), or a combination of these two forces.

Explanations involving decreased recombination rate per bp are based on the observation that most meiosis events have only 1–2 chiasmata per chromosome (Morescalchi and Galgano 1973; Pardo-Manuel de Villena and Sapienza 2001; Lynch 2007); thus, as genome size increases and the number of recombination events per chromosome remains constant, the recombination rate per bp should decrease (Lynch 2007; Lynch et al. 2011). Additionally, Haenel et al (2018) found a negative relationship between the average number of crossovers and the average chromosome length in animals, plants and fungi. Consequently, larger genomes with larger chromosomes would be expected to show low meiotic recombination rates per bp. This results in decreased *N*_*e*_ of linked loci and weakens the efficiency with which natural selection acts independently on individual loci (Hill and Robertson 1966). Insertions of DNA pose a mutational hazard because they have the potential to mutate to affect gene expression, intron splicing, or to generate harmful transcripts (Lynch et al. 2011). Therefore, longer stretches of DNA between recombination events likely mean stronger background selection in these regions, potentially decreasing the efficiency of selection in larger genomes.

The reduction in recombination rate per bp could also be a consequence, rather than a cause, of genome size growth. Genomes grow primarily by insertions of non-coding DNA, many of which may be only slightly deleterious (because of their mutational hazard) and can go undetected by natural selection when the *N*_*e*_ is small (Lynch and Conery 2003; Lynch et al. 2011). Thus, genetic drift in smaller populations could lead to larger genomes and lower recombination rates per bp, or the mechanisms of drift and weaker recombination could act together to allow nonadaptive genome expansion. While there is evidence that populations with smaller *N*_*e*_ do accumulate slightly deleterious mutations at a faster rate (Woolfit and Bromham 2005; Galtier 2016), Mohlhenrich and Mueller (2016) found no difference in strength of long-term drift between salamanders and frogs (the former of which have much larger genomes). Roddy et al. (2021) also found no relationship between genome size and census population size in mammals and argued that the mutational hazard hypothesis for genome size increase needs to be revisited.

Within vertebrates, salamanders are the clade with the widest variation in genome size and, except for a small number of lungfish species, the largest genomes. Most salamander families have evolved a wide range in genome size, indicating that genome size evolution has occurred independently in each of these lineages (Jockusch 1997). Decena-Segarra et al. (2020) and Segovia-Ramírez et al. (2023) estimated the genome size of 67 species of Neotropical plethodontid salamanders (tribe Bolitoglossini) comprising most of the variation in genome size in salamanders overall (9.8–87 pg). All bolitoglossine species have 13 pairs of chromosomes that are scaled versions depending on genome size (Sessions et al. 2008). Thus, if the number of crossover events per chromosome is constant in the group, genome size should scale linearly with recombination rate per bp across bolitoglossine species.

We take advantage of the wide variation in genome size within the Bolitoglossini and their karyotypic constancy to test the hypothesis that larger genomes have less efficient natural selection, either because of lower recombination rate per bp that leads to stronger linked selection or because of an association between large genome size and small effective population size in these species leading to stronger drift and less efficient selection. We test for both elevated values of d*N*/d*S* in species with larger genomes and relaxed selection in these species using a transcriptomic dataset. Finally, we use a proxy of effective population size (area of species’ geographic distribution) to test for a relationship between genome size and effective population size.

## Materials and Methods

For the 15 species dataset (15-spp. set or full dataset, hereafter) we chose 15 species from six different genera of bolitoglossine salamanders (*Bolitoglossa, Chiropterotriton, Dendrotriton, Parvimolge, Pseudoeurycea*, and *Thorius*) using genome size as the principal criterion for selection. We used genome size estimations from Decena-Segarra et al. (2020) and Segovia-Ramírez et al. (2023) to select bolitoglossine species with small (12.4–30.7 pg) and large genomes (54.6–87.2 pg). To increase the number of orthologs in our analysis, we used a second dataset with only six of the 15 species (*Thorius* sp. “San Juan del Estado”*, Chiropterotriton orculus, Parvimolge townsendi, Bolitoglossa franklini, B. stuarti and B. macrini*) following Mohlhenrich and Mueller (2016) and conducted all analyses on both the full dataset and the six-species dataset. This subset of six species (six-spp. set hereafter) included the largest (74.2–87.2 pg) and smallest (12.4–20.6 pg) genome sizes of the 15-spp. set. These species were collected between 2015 and 2018 in Mexico. Each salamander was anesthetized with MS-222 and the spleen, heart, liver, and intestine were removed and preserved in NAP buffer (Camacho-Sanchez et al. 2013). In most cases, NAP-preserved samples were kept in liquid nitrogen after 24 h until arrival at the lab, where they were transferred to – 80 °C.

Because our aim was to obtain the largest number of transcripts for each species, we extracted RNA using TRIzol (Invitrogen, Carlsbad, CA) from the four tissue types and pooled extracted RNA for each salamander. Prior to library preparation, we ran pooled RNA for each species on a Bioanalyzer to estimate RNA quality. We used a modified protocol for Illumina TruSeq RNA library Prep v2 (Illumina, San Diego, California) for transcriptome libraries. In brief, modifications consisted in performing half reactions for all steps in the protocol to minimize the amount of RNA needed for library construction because of limited tissue quantity for some species of miniature salamanders. We quantified library quality and concentration using an Agilent 2100 Bioanalyzer system and Qubit 3.0 (Thermo Fisher Scientific) for use in pooling. Thirteen of the fifteen species used in this study were sequenced on a single HiSeq 4000 lane (Illumina) to generate 100 pb PE reads and were used in the study of Segovia-Ramírez et a. (2023). The remaining two (together with libraries from a separate project) were sequenced on one lane of an Illumina Novaseq 6000 150 PE at the QB3 Vincent J Coates Genomics Sequencing Lab, University of California, Berkeley.

The 15 species were analyzed using a pipeline of Singhal (2013) for quality evaluation, read cleaning, assembly, annotation and ortholog alignment. Specifically, the quality of the raw reads was assessed with FASTQC v0.1.1.5 (Andrews 2012); duplicates, low-quality reads, and adapter sequences were removed with TRIMMOMATIC v0.39 (Lohse et al. 2012) and cutadapt v1.18. Reads that matched contaminant sources (rRNA or bacterial sources) were removed with BOWTIE2 v2.3.5 with default parameters (Langmead and Salzberg 2012) and overlapping paired reads were spliced and aligned with FLASH v1.2.11 (Magoč and Salzberg 2011) and COPE v1.1.2.

We used TRINITY v2.14.0 (Grabherr et al. 2011) for de novo assembly of each transcriptome and removed redundancies with standard parameters of CD-HIT-EST v4.7 (Li and Godzik 2006). To annotate each transcriptome, we used a reciprocal best-match approach using BLASTX v2.6.0 (Altschul et al. 1997). Coding and non-coding regions were defined with EXONERATE v2.4.0 (Slater and Birney 2005), and FRAMEDP v1.2.2 was used to identify frameshift mutations (Gouzy et al. 2009). *Xenopus tropicalis*, the best annotated amphibian genome, and three other well annotated vertebrate genomes (*Anolis carolinensis*, *Mus musculus* and *Homo sapiens*) from the Ensembl protein database (Cunningham et al. 2022) were used as references for annotation. Subsequently, Perl pipelines were used to discard redundant annotated transcripts and retain only those unique by species by means of self-blasting against all references.

For ortholog alignment, we used getorf EMBOSS v6.6.0 (Rice et al. 2000) to find and generate sequences with open reading frames, PRANK v150803 (Löytynoja and Goldman 2010) to perform a codon-based alignment and Gblocks v0.91b (Talavera and Castresana 2007) to eliminate misaligned positions with missing data or ambiguous gaps. We retained only those alignments with a length > 150 nucleotides, that is, 50 amino acids (Künstner et al. 2010), and a transition/transversion ratio (t*s*/t*v*) < 1 (Yang and Nielsen 2000).

To estimate differences in efficiency of selection, we used a concatenated alignment of the filtered genes and an ultrametric phylogeny estimated from mtDNA and five nuclear genes from Decena-Segarra et al. (2020), which we pruned to include only the species in our study. We estimated d*N*/d*S* (*ω*) using the branch models implemented in the CodeML program of PAML version 4.9 (Yang 2007) as described in Jeffares et al. (2015). CodeML works by calculating the fit of a specified model of evolution to a phylogenetic data set and uses likelihood ratio tests to assess which model provides the best fit to the data. PAML has been widely used to analyze protein-coding gene sequences to estimate the synonymous and nonsynonymous rates (d*S* and d*N*) and to detect positive selection driving protein evolution (Álvarez-Carretero et al. 2023).

We first ran CodeML’s free-ratio branch model to estimate a single value of d*N*, d*S* and *ω* for each lineage on our unrooted phylogeny (set in control CodeML file as NSsites = 0, model = 1). Then, to study if the species with larger genomes are more likely to have different *ω* than the species with smaller genomes, we ran the one-ratio and two-ratio models using the concatenated set of genes. The one-ratio model is used as a null model with one average omega for all branches of the phylogeny (set in control CodeML file as NSsites = 0, model = 0). As an alternative model, we used the two- ratio model that allows different *ω* values for test, reference, and internal branches (set in control CodeML file as NSsites = 0, model = 2). We classified all species with genomes below 31 pg as the reference set and species with larger genomes (> 54 pg) as the test set. Although the values used to define these groups were arbitrary, our aim is to compare larger genomes against smaller ones using two groups with contrasting genome sizes. Additionally, we analyzed each ortholog individually to assess the number of genes with different *ω* values between the test and reference branches. Higher *ω* values for the species with larger genomes in the two-ratio branch model would support our hypothesis that salamanders with larger genomes accumulate deleterious mutations faster than salamanders with smaller genomes (Neiman et al. 2010). We repeated these analyses for the concatenated set of orthologs and individually for each ortholog of the six-spp. dataset.

If the salamanders with larger genomes have a higher *ω* than salamanders with smaller genomes, this could be due to either more widespread positive selection (*ω* > 1) in species with larger genomes or relaxed purifying selection in those species. To distinguish between these two scenarios, we used RELAX (Wertheim et al. 2015) in the Hypothesis Testing using Phylogenies (HyPhy) version 2.3 software package (Pond et al. 2005). RELAX tests whether the strength of selection has been intensified or relaxed along a specified set of test branches by estimating an exponent (*k*) of the *ω* values for sites under purifying (*ω* < 1) and positive (*ω* > 1) selection in the test set. Values of *k* > 1 indicate intensification of selection in the test set while *k* < 1 indicates relaxation of selection in the test set. We labeled the branches as described above, with the species with smaller genomes as the reference set and the species with larger genomes as the test set. Lastly, we analyzed each ortholog individually to assess the number of genes under relaxation or intensification for the six-spp. set and 15-spp. sets.

Selection efficiency is predicted to be lower in small populations because slightly deleterious mutations may be fixed by drift at small *N*_*e*_ (Ohta 1973). While small *N*_*e*_ is hypothesized to increase genome size via fixation of slightly deleterious insertions (Lynch 2007), Mohlhenrich and Mueller (2016) found no relationship between the strength of genetic drift and genome size in salamanders and frogs. The ecology and natural history of the species in our study is relatively poorly known and no estimates of census population size (*N*_*c*_) or* N*_*e*_ exist for any of them. To test for relationship of population size with genome size and *ω*, we used estimates of geographic range size from Segovia-Ramírez et al. (2023) as a proxy of population size. Briefly, these estimates were obtained by adding a 10 km buffer around all museum localities for each species obtained from the Global Biodiversity Information Facility (www.gbif.org) and merging buffers to obtain an estimate of the species’ range. We tested for a relationship between genome size and range size, and *ω* and range size using simple linear regression with the ‘cor.test’ function and phylogenetic independent contrast with the ‘lm’ function from ape v5.0 package (Paradis and Schliep 2019) in R. We also use estimates of geographic range size from an additional 39 species, for a total of 54 species of 11 bolitoglossine genera with genome size estimates to test for a correlation with genome size and geographical range size more broadly in this group of salamanders (Online Resource 1).

## Results

We obtained between 4997 and 9654 annotated transcripts with an average length of 563–887 bp for the species in our study (Table 1). For the 15-spp. set, we retained 207 orthologs with a concatenated length of 62358 bp (20,786 codons) and for the six-spp. set we retained 763 orthologs with a concatenated length of 260,355 bp (86785 codons).

**Table 1:** Summary of de novo transcriptome assembly metrics for the fifteen species included in the study

Species	Total length	Total contigs	Mean	Median	Max	N50	N90	GC%
*Bolitoglossa franklini*	3380073	5996	563	438	3873	693	282	48.35
*Bolitoglossa macrinii*	8571315	9654	887	708	8733	1155	447	47.68
*Bolitoglossa riletti*	6137112	8457	725	591	5871	924	372	47.25
*Bolitoglossa hartwegi*	4108461	6237	658	501	8055	849	324	47.34
*Bolitoglossa stuarti*	5022681	7317	686	543	6489	885	339	47.74
*Bolitoglossa mombachoensis*	3591801	5460	657	492	13857	861	318	47.27
*Pseudoeurycea juarezi*	5525307	7875	701	555	7701	888	357	48.28
*Parvimolge townsendi*	6774897	8507	796	648	9303	1026	405	47.46
*Chiropterotriton lavae*	5535267	7885	701	555	7809	897	354	48.24
*Chiropterotriton orculus*	6514764	8603	757	603	6336	969	387	47.75
*Chiropterotriton dimidiatus*	5525310	7534	733	588	7737	948	372	47.68
*Dendrotriton xolocalcae*	3078450	4997	616	459	13848	750	315	48.13
*Thorius lunaris*	4807272	7169	670	528	7422	861	336	48.00
*Thorius spilogaster*	5778666	8024	720	573	6042	921	363	48.35
*Thorius* sp. “San Juan del Estado”	6594429	9231	714	573	5826	903	366	47.74

Mean, median, and Max refer to contig length

The first seven species have larger genomes (C-value > 54 pg) and the following eight species have smaller genomes (C-value < 31 pg)

**Fig. 1:**
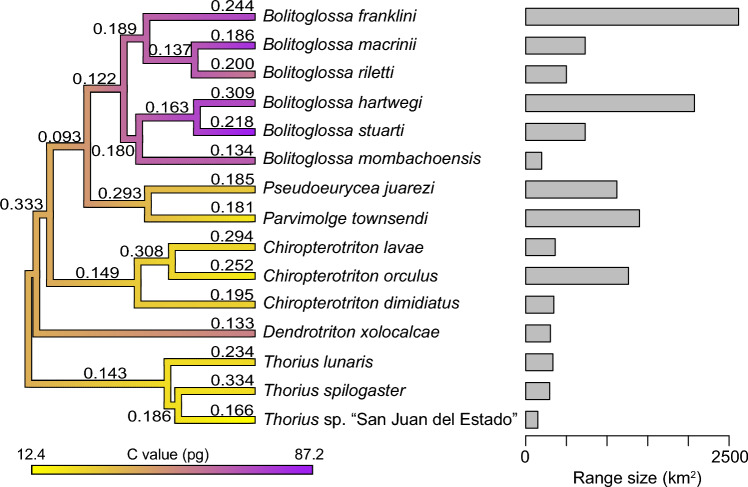
Phylogenetic tree of species used in study and range size estimation. Branch-specific selective pressure and range size estimated from 207 orthologous genes in 15 Neotropical salamanders. Branches are colored by genome size, with genome size estimations for internal branches estimated using the countMap function of phytools (Revell 2012). Numbers on each branch are estimates of *ω* from the CodeML free ratio model

The free-ratio model estimated *ω* values between 0.1338 and 0.3337 for species in the 15-spp. set and 0.1255–0.2237 (Fig. 1, Table 2) for the six-spp. set (Table 3). We find no significant relationship between *ω* and genome size for either set (15-spp.: *r* =  − 0.11, *p* = 0.69; six-spp.: *r* = 0.2, *p* = 0.72).

**Table 2:** Estimates of the rate of non-synonymous substitutions per non-synonymous site (d*N*), synonymous substitutions per synonymous site (d*S*), and d*N*/d*S* (*ω*) from the free-ratio model for the 15-spp. set

Species	d*N*	d*S*	d*N*/d*S* (ω)
*Bolitoglossa franklini*	0.0099	0.0406	0.2445
*Bolitoglossa macrinii*	0.0038	0.0207	0.1861
*Bolitoglossa riletti*	0.0033	0.0166	0.2004
*Bolitoglossa hartwegi*	0.0063	0.0205	0.3092
*Bolitoglossa stuarti*	0.0034	0.0157	0.2188
*Bolitoglossa mombachoensis*	0.0070	0.0521	0.1348
*Pseudoeurycea juarezi*	0.0092	0.0500	0.1858
*Parvimolge townsendi*	0.0164	0.0909	0.1811
*Chiropterotriton lavae*	0.0047	0.0160	0.2943
*Chiropterotriton orculus*	0.0053	0.0211	0.2526
*Chiropterotriton dimidiatus*	0.0058	0.0297	0.1956
*Dendrotriton xolocalcae*	0.0189	0.1411	0.1339
*Thorius lunaris*	0.0021	0.0092	0.2340
*Thorius spilogaster*	0.0034	0.0102	0.3341
*Thorius* sp. “San Juan del Estado”	0.0207	0.1546	0.1664

The first seven species have larger genomes (C-value > 54 pg) and the following eight species have smaller genomes (C-value < 31 pg)

**Table 3:** Estimates of the rate of non-synonymous substitutions per non-synonymous site (d*N*), synonymous substitutions per synonymous site (d*S*), and  d*N*/d*S* (*ω*) from the free-ratio model for the six-spp. set

Species	d*N*	d*S*	d*N*/d*S* (ω)
*Bolitoglossa franklini*	0.0080	0.0359	0.2237
*Bolitoglossa macrini*	0.0061	0.0437	0.1405
*Bolitoglossa stuarti*	0.0088	0.0567	0.1567
*Parvimolge townsendi*	0.0128	0.0924	0.1386
*Chiropterotriton orculus*	0.0202	0.1608	0.1255
*Thorius* sp. “San Juan del Estado”	0.0139	0.1041	0.1319

Analyzing each ortholog separately, a single rate model fits better for 80.2% (166 of 207) of orthologs in the 15-spp. Set (Table 4). For the 41 orthologs with a better fit of a two-ratio model, 9.7% (20/207) have a higher *ω* for species with smaller genomes and 10.1% (21/207) have a higher *ω* values in the species with larger genome sizes, with very similar average *ω* values in both cases. The six-spp. set shows a similar percentage of orthologs (84.5%, 645 of 763) for which the one-rate model fits better (Table 4). For the remaining orthologs, only 1.8% (14/763) have a higher ω in species with smaller genomes while 13.6% (104/763) have a higher *ω* in the species with larger genomes. As in the full dataset, the average *ω* for both groups of species is very similar.

**Table 4:** Results from the one-*ω* and two-*ω* models  for orthologs for the 15-spp and six-spp. set

	Six-spp. set	15-spp. set
Number of orthologs	763	207
One-ω model	84.53% (645/763)	80.19% (166/207)
Two-ω model Sω > Lω	1.83% (14/763)	9.66% (20/207)
Two-ω model Sω < Lω	13.63% (104/763)	10.14% (21/207)
Median Sω	0.090 (0.037, 0.181)	0.107 (0.037, 0.231)
Median Lω	0.104 (0.042, 0.222)	0.104 (0.033, 0.202)

The proportion of genes for which a two-ratio model fits better is given, with the number of genes in parenthesis

For estimated *ω* values, the median is shown with 25 and 75% quantiles in parentheses

L: group of species with larger genome sizes

S: group of species with small genome sizes

The results of the RELAX analysis of each ortholog in the 15-spp. set shows a similar number of genes under relaxation (10, median *k* = 0.14) and intensification (nine, median *k* = 28.20) in the species with large genomes compared to those with small genomes (Table 5). By contrast, for the six-spp. set analysis there are more genes under intensification (40, median *k* = 3.03) than under relaxation (18, median *k* = 0.25) in the species with large genomes (Table 5).

**Table 5:** Results of RELAX analyses

	Six-spp. set	15-spp. set
Genes under intensification	5.24% (40/763)	4.34% (9/207)
Genes under relaxation	2.35% (18/763)	4.83% (10/207)
Median k of genes under intensification	3.038	28.201
Median k of genes under relaxation	0.250	0.143

The number of genes under relaxation or intensification and the total number of genes analyzed are shown in parenthesis

Median *k* values refer to the exponent applied to *ω* values, with *k* > 1 corresponding to selection intensification and *k* < 1 corresponding to relaxation of selection

**Fig. 2:**
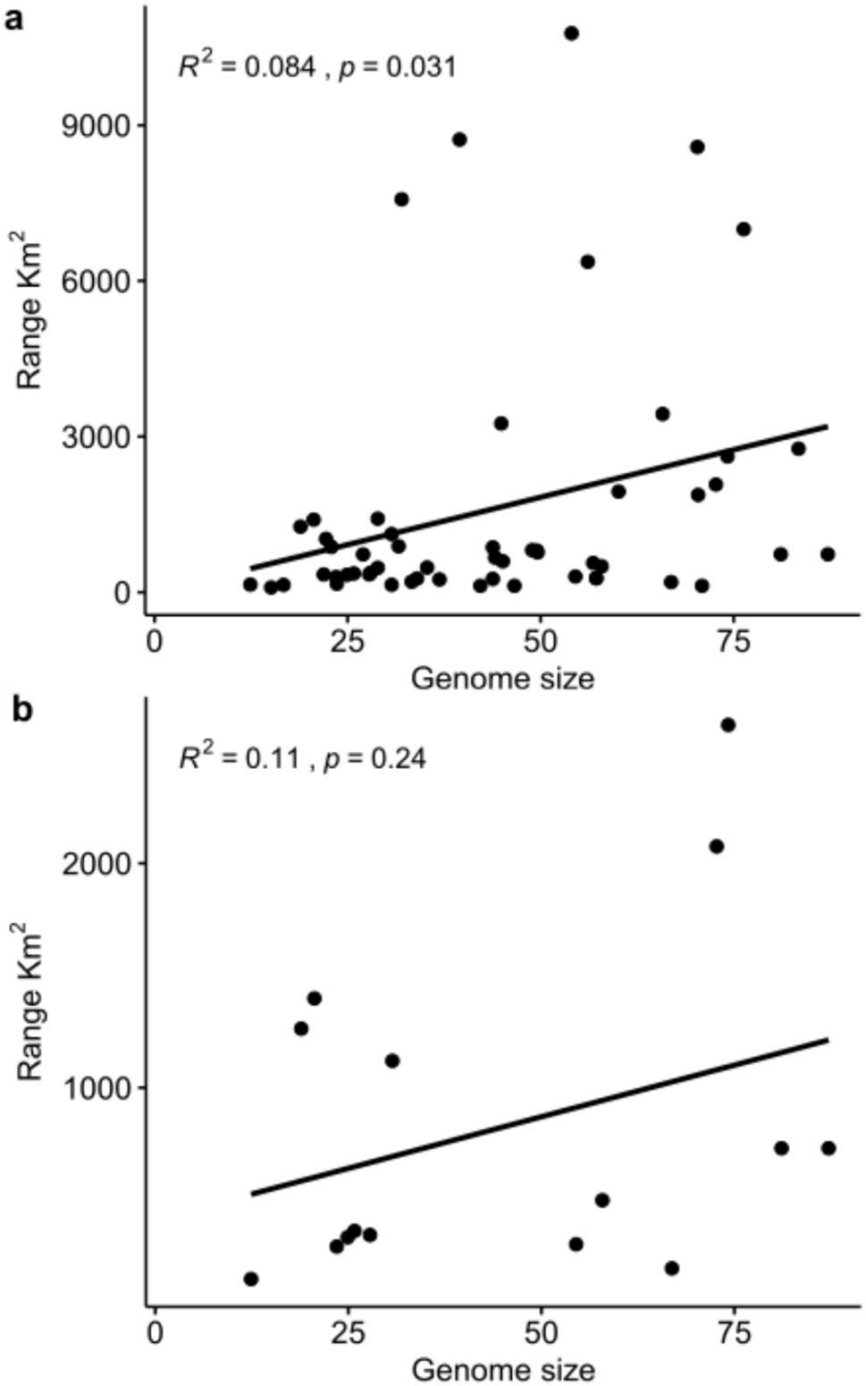
Correlations of genome size and geographical range size (km^2^). **a** Significant positive correlation using 54 species of Bolitoglossini **b** Nonsignificant positive correlation using only 15 species included in this study

Finally, we found a positive but non-significant relationship between genome size and geographic range size for the 15 species in our study (*R*^2^ = 0.11, *p* = 0.24) but did find a significant positive correlation using the expanded set of 54 species (*R*^2^ = 0.084, *p* = 0.03) (Fig. 2). The phylogenetic generalized least squares regression for the set of 15 and 54 species was not significant (*F* = 0.007, *p* = 0.93; *F* = 0.845, *p* = 0.36, respectively). Neither ordinary regression nor PGLS found a significant relationship between range size and d*N*/d*S* for the 15 species data set (OLS: *R*^2^ = 0.11, *p* = 0.24; PGLS: *F* = 3.88, *p* = 0.07).

## Discussion

We hypothesized that genome size would be negatively correlated with efficiency of selection and that species with larger genomes would show relaxation of natural selection, either because of decreased recombination rate per bp in larger chromosomes or because of smaller effective population size that results in genomic expansion and fixation of slightly deleterious mutations via increased genetic drift. Instead, we found no significant relationship between *ω* estimates and genome size using the free ratio model in CodeML. Most genes in both datasets showed a better fit of a model with a single *ω* value for species with small and large genomes, and for the remaining genes only the six-spp. set showed more orthologs with higher *ω* values in species with large genomes. Results of the RELAX analyses revealed only a small proportion of the total orthologs under relaxed or intensified selection in both datasets, with more genes under intensification in the species with large genomes for the six-spp. set, contrary to our expectations. Taken together, none of these analyses provides strong support for our hypothesis, and instead they indicate that the efficiency of selection appears to be similar between species with large and small genomes.

Our estimates of *ω* from the free-ratio model (0.13–0.33 for the 15-spp. set and 0.12–0.22 for the six-spp. set, Tables 2 and 3) are relatively high compared to those reported for most other vertebrates. Toll-Riera et al. (2011) reported a *ω* range of 0.098–0.163 for 2929 genes from eight species of mammals. Weber et al. (2014) analyzed 921 genes of 48 species of birds and estimated a range of *ω* of 0.13–0.17 while Yang et al. (2016) analyzed 5107 genes from two species of frogs and reported *ω* values of 0.11–0.13. Mohlhenrich and Mueller (2016) estimated median *ω* values of 0.048 using six salamander species and 0.060 using three salamander species but did not use a free-ratio model to estimate *ω* values for each species separately. Similar to our results, Fuselli et al. (2023) estimated *ω* values of 0.141–0.332 for five species of lungfish, the vertebrates with the largest genomes. The higher *ω* values of the lungfish and those that we report are broadly in agreement with the idea that selection may be relaxed in species with larger genomes, but the values reported by Mohlhenrich and Mueller (2016) do not follow the same pattern.

Of all our results from CodeML, the only result that agrees with our predictions is the higher number of genes from species with larger genomes having a better fit of a two-rate model with a higher *ω* (Sω < Lω); the rest of the results suggest no difference in selection efficiency between groups. Mohlhenrich and Mueller (2016) found a similar result in their comparison of frogs and salamanders. For a set of 12 species (six frogs and six salamanders) they found similar median *ω* values for each group (0.046 for frogs and 0.048 for salamanders) and a similar proportion of genes for which a two-ratio model fit better (higher *ω* in frogs for 18% of genes and higher *ω* for salamanders in 15% of genes). In a reduced set of six species (three frogs and three salamanders), they again found similar median *ω* values between both groups (0.054 for frogs and 0.060 for salamanders) and a higher number of genes for which a model with a larger major *ω* fit better in salamanders (21.1%) than in frogs (9.3%). They concluded that drift does not seem to be stronger in salamanders than in frogs, and our results agree broadly with the conclusion that drift doesn't seem to be stronger in species with large genomes.

The results of RELAX do not show a clear pattern of relaxation of natural selection in the species with larger genomes, contrary to our expectations; in fact, we find more genes with significant intensification of selection than those with relaxation in the six-spp. set. A recent study used both CodeML and RELAX to test for a relaxation of selection in lungfish genomes and found 139 and six genes under relaxed and intensified selection, respectively, compared to other tetrapods (Fuselli et al. 2023). This study included several salamander species and found that, contrary to expectations of stronger drift in larger genomes, the salamander transcriptomes behaved similarly to other non-lungfish tetrapods in terms of relaxation of selection. Thus, salamanders do not seem to have a signature of relaxed selection and stronger drift despite their large genome sizes, and this trend holds within the group of salamanders with the widest variation in genome size based on our results.

Estimates of *ω* for a group of isopods that underwent repeated transitions to subterranean environments with a hypothesized accompanying decrease in population sizes showed increased *ω*, increased genome size, and decreased values of theta (product of effective population size and mutation rate) in subterranean species (Lefébure et al. 2017). Unlike our results, all of these agree with the hypothesis of relaxed selection and increased genome size in smaller populations, although the genome sizes of these isopods (< 3 Gb) are substantially smaller than those in our study. The discrepancy between results in plethodontid salamanders (Mohlhenrich and Mueller 2016, this study) and those in lungfish and isopods argue against a general pattern of reduced selection efficiency in species with larger genomes because of drift in small populations. If there is a general pattern, however, salamanders are an outlier, as in so many other aspects of their biology (Sessions and Wake 2021; Yun 2021).

The lack of a relationship between genome size and selection efficiency in this group could be a result of methodological or biological considerations. Although no direct estimates of census or effective population size are available for the species in our study, one proxy of census population size (range size, Gaston and Blackburn 1996) shows a positive relationship with genome size in bolitoglossines, including a positive (but nonsignificant) relationship in the 15 species in our study. If range size is an accurate census proxy of population size, this also argues against a primary role for drift in increasing genome size in this group, in agreement with our results. It is important to note, however, that many factors including demographic fluctuations, differences in sex ratios or reproductive success among individuals, and population subdivision can alter the ratio of census population size to effective population size (*N*_*c*_/*N*_*e*_) and that these factors likely do not impact all species equally. Furthermore, *N*_*c*_ primarily affects the number of mutations that arise in a population and is thus a forward-looking measure while *N*_*e*_ affects the fixation of these mutations in a population and is thus a backward-looking measure (Platt et al. 2018). Because we have no estimate of *N*_*e*_ and only a proxy of *N*_*c*_, conclusions from the relationship between range size and genome size should be viewed with caution.

A positive relationship between genome size and body size also exists, in agreement with smaller population size in species with large genomes (as body size and population size are expected to be negatively correlated); this may be due to selection for smaller genomes in miniaturized species, however, rather than drift (Decena-Segarra et al. 2020). Finally, levels of genetic polymorphism within species are not correlated with genome size for either transcriptomic loci or putatively noncoding (ddRAD) loci, and a negative correlation between genetic diversity and genome size would be expected if smaller population size led to larger genome sizes (Segovia-Ramírez et al. 2023). Thus, all results to date argue that salamanders have not experienced stronger long-term drift resulting in larger genomes and reduced selection efficiency.

The hypothesis of reduced selection efficiency resulting from a reduction in per bp recombination rate rests on the assumption that genome size is negatively correlated with recombination rate because of a nearly constant number of chiasmata per chromosome arm. There is some evidence that the number of chiasmata per chromosome in plethodontid salamanders may be higher than the 1–2 observed in most other vertebrates (Stanley Sessions, unpublished data; Morescalchi and Galgano 1973; Macgregor 1993; Pardo-Manuel de Villena and Sapienza 2001). If the number of chiasmata increases with genome size in this group, then there would not necessarily be a relationship between genome size and recombination rate per bp, eliminating the expectation of stronger linked selection and a reduction in selection efficiency in species with larger genomes.

Like other groups with genomic gigantism, salamander genomes have grown large through the activity of transposable elements (Sun et al. 2012). If selection efficiency is not reduced in species with large genomes, another explanation must be sought for the fixation of so many seemingly nonfunctional elements in salamander genomes. Adaptive explanations of genome size increase provide alternative hypotheses to be tested.

### Supplementary Information

Below is the link to the electronic supplementary material.

Supplementary file1 (DOCX 19 KB)

## Data Availability

Illumina sequence data for transcriptomes of 15 species is deposited in the NCBI Short Read Archive (BioProjects PRJNA1023252, PRJNA1076578). Ortholog alignments for the 15-spp and six-spp datasets and the phylogenetic tree used in analyses are deposited in the Zenodo repository at the URL: 10.5281/zenodo.10641326.
