# Supplementary material for: Genomic Gigantism is not Associated with Reduced Selection Efficiency in Neotropical Salamanders
Source: J Mol Evol. 2024 Jun 6;92(4):371–80. doi: 10.1007/s00239-024-10177-w (PMC11291587; doi:10.1007/s00239-024-10177-w)
Supplement: Supplementary file 1 — Supplementary file1 (DOCX 19 KB) [file 239_2024_10177_MOESM1_ESM.docx]

**Genomic gigantism is not associated with reduced selection efficiency in Neotropical salamanders**

Hairo Rios-Carlos^1^, María Guadalupe Segovia-Ramírez^1^, Matthew K. Fujita^2^, Sean M. Rovito^1, 3^

^1^Unidad de Genómica Avanzada, Centro de Investigación y de Estudios Avanzados del Instituto Politécnico Nacional, km 9.6 Libramiento Norte Carretera Irapuato-León, Irapuato, Guanajuato, México

^2^ Amphibian and Reptile Diversity Research Center, Department of Biology, The University of Texas, Arlington, Texas, USA

^3^Address for correspondence: [sean.rovito@cinvestav.mx](mailto:sean.rovito@cinvestav.mx)

Online Resource 1. Range Size estimation for the set of 54 species and their C values. The last 15 species are those included in this study.

| **Species** | **C value (pg)** | **Range (Km^2^)** |
| --- | --- | --- |
| *Aquiloeurycea quetzalanensis* | 35.3 | 476.09 |
| *Aquiloeurycea scandens* | 49.5 | 802.46 |
| *Aquiloeurycea* sp. “Qro” | 57.2 | 270.69 |
| *Bolitoglossa chinanteca* | 43.8 | 256.43 |
| *Bolitoglossa flaviventris* | 70.4 | 1875.37 |
| *Bolitoglossa insularis* | 70.9 | 123.40 |
| *Bolitoglossa lincolni* | 83.4 | 2766.47 |
| *Bolitoglossa mexicana* | 76.3 | 6995.27 |
| *Bolitoglossa occidentalis* | 56.1 | 6367.08 |
| *Bolitoglossa platydactyla* | 70.3 | 8579.11 |
| *Bolitoglossa rufescens* | 39.5 | 8722.40 |
| *Bolitoglossa striatula* | 65.8 | 3433.65 |
| *Bolitoglossa zapoteca* | 42.2 | 124.06 |
| *Chiropterotriton chico* | 28 | 377.05 |
| *Chiropterotriton magnipes* | 27 | 729.04 |
| *Chiropterotriton miquihuanus* | 33.3 | 202.97 |
| *Chiropterotriton terrestris* | 21.9 | 342.43 |
| *Chiropterotriton alvarezdeltoroi* | 30.7 | 143.49 |
| *Dendrotriton bromeliacius* | 36.9 | 246.88 |
| *Dendrotriton megarhinus* | 46.6 | 124.03 |
| *Isthmura bellii* | 54 | 10770.61 |
| *Isthmura gigantea* | 43.8 | 863.74 |
| *Nyctanolis pernix* | 45.1 | 596.05 |
| *Oedipina uniformis* | 44.9 | 3252.09 |
| *Pseudoeurycea altamontana* | 49.6 | 766.24 |
| *Pseudoeurycea conanti* | 34 | 265.72 |
| *Pseudoeurycea gadovii* | 28.9 | 1417.68 |
| *Pseudoeurycea leprosa* | 32 | 7572.08 |
| *Pseudoeurycea lineola* | 31.6 | 885.375 |
| *Pseudoeurycea longicauda* | 44.1 | 668.05 |
| *Pseudoeurycea melanomolga* | 56.8 | 563.29 |
| *Pseudoeurycea robertsi* | 48.9 | 811.68 |
| *Thorius aureus* | 16.7 | 141.71 |
| *Thorius* sp. “Ixtlan” | 15.1 | 92.38 |
| *Thorius narisovalis* | 22.9 | 872.10 |
| *Thorius* sp. 7 | 23.6 | 162.53 |
| *Thorius tlaxiacus* | 28.9 | 470.29 |
| *Bolitoglossa franklini* | 74.2 | 2616.31 |
| *Bolitoglossa hartwegi* | 72.7 | 2075.17 |
| *Bolitoglossa macrinii* | 81.1 | 730.55 |
| *Bolitoglossa mombachoensis* | 66.9 | 195.65 |
| *Bolitoglossa riletti* | 57.9 | 499.15 |
| *Bolitoglossa stuarti* | 87.2 | 730.448 |
| *Bolitoglossa yucatana* | 60.1 | 1939.97 |
| *Chiropterotriton dimidiatus* | 27.8 | 344.419 |
| *Chiropterotriton lavae* | 25.8 | 362.56 |
| *Chiropterotriton orculus* | 18.9 | 1263.56 |
| *Dendrotriton xolocalcae* | 24.5 | 302.96 |
| *Parvimolge townsendi* | 20.6 | 1398.12 |
| *Pseudoeurycea juarezi* | 30.7 | 1120.13 |
| *Thorius lunaris* | 24.9 | 334.154 |
| *Thorius pulmonaris* | 22.2 | 1028.69 |
| *Thorius* sp. “San Juan del Estado” | 12.4 | 148.114 |
| *Thorius spilogaster* | 23.5 | 293.08 |
